# Diagnostic Accuracy of 18F-FDG-PET/CT and MRI in Predicting the Tumor Response in Locally Advanced Cervical Carcinoma Treated by Chemoradiotherapy: A Meta-Analysis

**DOI:** 10.1155/2021/8874990

**Published:** 2021-03-02

**Authors:** Sharareh Sanei Sistani, Fateme Parooie, Morteza Salarzaei

**Affiliations:** ^1^Department of Radiology, School Medicine, Zahedan University of Medical Science, Zahedan, Iran; ^2^Student Research Committee, Zabol University of Medical Science, Zabol, Iran

## Abstract

**Objective:**

The aim of this meta-analysis was to compare the diagnostic accuracy of 18F-FDG-PET/CT and MRI in predicting the tumor response in locally advanced cervical carcinoma (LACC) treated by chemoradiotherapy (CRT).

**Method:**

This meta-analysis has been performed according to PRISMA guidelines. Systematic searches were conducted using PubMed and Embase databases for articles published from January 1, 2010, to January 1, 2020. By using the Quality Assessment of Diagnostic Accuracy Studies 2 (QUADAS-2) tool, the reviewers assessed the methodological quality scores of the selected studies. We analyzed the sensitivity, specificity, and accuracy of two diagnostic methods using Meta-DiSc 1.4 and Stata 15.

**Results:**

An overall of 15 studies including 1132 patients were included. Sensitivities of PET/CT and MRI were 83.5% and 82.7%, while the corresponding rates for specificities were 77.8% and 68.4%, respectively. The DOR, PLR, and NLR for MRI were 15.140, 2.92, and 22.6. PET/CT had a DOR of 25.21. The PLR and NLR for PET/CT were 4.13 and 0.215, respectively. The diagnostic sensitivity and specificity of PET/CT for the detection of residual tumor were 86% and 95%, respectively. The corresponding rates for MRI were 73% and 96%, respectively. The diagnostic sensitivity and specificity of PET/CT for the detection of tumor metastases were 97% and 99%, while the corresponding rates for MRI were 31% and 98%, respectively.

**Conclusion:**

18F-FDG PET/CT seemed to have a better overall diagnostic accuracy in the evaluation of treatment response to chemoradiotherapy in LACC patients. MRI showed a really poor sensitivity in the detection of metastases, and PET/CT performed significantly better. However, the difference between these two methods in the detection of residual disease was not significant. More studies are needed to be conducted in order to approve that 18F-FDG PET/CT can be a standard option to assess the treatment response.

## 1. Introduction

Cervical cancer is one of the most prevalent cancers among women [1]. About 40% of these patients show locally advanced cervical carcinoma (LACC) at initial diagnosis [2]. The standard treatment for LACC is specific cisplatin-based radiotherapy chemotherapy (CRT) [3]. However, usually, in 33% of cases, the tumor recurs about 2 years after CRT, and the overall 5-year survival is approximately 70% [3]. Lymph node status, response to treatment, and clinical stage are the main predictors of recurrence. Among the new strategies, neoadjuvant CRT followed by radical surgery is performed with the purpose of removing residual tumors which are potentially radio- and chemo-resistant and also improving local control and survival [4,5]. In addition, radical surgery provides useful prognostic information, which is the pathological response to treatment. Women who obtain a complete pathological response to neoadjuvant CRT showed significantly longer overall survival and disease-free survival than women with partial response [6,7]. However, applying this approach has some intraoperative and postoperative complications [8]. Therefore, there is a need for imaging techniques that can evaluate the tumor response accurately during and after treatment, so personalized treatment can be made possible. Imaging techniques such as magnetic resonance imaging (MRI), computed tomography (CT), and positron emission tomography (PET) with 18F-fluorodeoxyglucose (FDG) are more useful tools for evaluating how extensive the tumor is [9]. In the evaluation of tumor size and invasion and the local extent of the disease, MRI is superior to CT. Unlike CT and MRI, PET is used to evaluate tumor metabolic function. Pathological tumor size in cervical cancer and tumor size measured by PET have been shown to have strong correlation [10]. PET has been used to evaluate pretreatment situation and daily surveillance of patients with cervical cancer after treatment [11]. 3-month posttreatment PET can be applied to predict the therapeutic response [12]. However, patients who have a poor response to CRT can be detected as soon as possible, and their treatment plan can be changed as well, provided that the tumor response can be estimated during or just after concomitant chemoradiotherapy. 18F-FDG-PET/CT is important in LACC staging due to its capability to detect distant metastases and involved lymph nodes; it, therefore, improves treatment planning [13]. Furthermore, 18F-FDG-PET/CT can predict better prognosis in patients who developed complete metabolic response when performed after exclusive CRT [13]. In contrast, the role of 18F-FDG-PET/CT performed during treatment is not defined apparently [4]. The aim of this meta-analysis is to compare the diagnostic accuracy of 18F-FDG-PET/CT and MRI in predicting the tumor response in locally advanced cervical carcinoma treated by chemoradiotherapy.

## 2. Materials and Methods

### 2.1. Literature Search

This meta-analysis has been performed according to Preferred Reporting Items for Systematic Reviews and Meta-Analyses guidelines [14]. Systematic searches were conducted using PubMed and Embase databases for articles published from January 1, 2010, to January 1, 2020. The search query was done using the key terms “cervical cancer,” “magnetic resonance imaging or MRI,” “18F-FDG PET/CT,” “accuracy,” “specificity,” “sensitivity,” and “prognosis,” and related terms are as follows: (cervix or cervical) and (PET or positron emission tomography) or (FDG or fluorodeoxyglucose). There was no limitation regarding the language of the studies. The bibliography of retrieved early studies was cross-examined in order to find other related papers and articles.

The inclusion criteria consisted of the following: studies evaluating PET/CT and MRI diagnostic accuracy in the treatment of LACC. Pathological results were considered as the “gold standard.” The diagnostic criteria were as follows: there are 2 × 2 contingency tables (Table 1); direct or indirect access to true positive, false positive, true negative, false negative, specificity, and sensitivity. We also included some additional studies as they provided data which helped to complete our manuscript and make it more understandable. These data were analyzed separately from the diagnostic accuracy data. The exclusion criteria were as follows: publication type other than the authentic research papers (i.e., review articles and conference abstracts), not in the field of the researchers' interest. The searching process and selection of the articles were done by two independent reviewers with 3 years of meta-analysis experience. Disagreements were resolved through discussion.

### 2.2. Data Extraction and Quality Assessment

The two abovementioned authors separately assessed each study and then extracted the data by applying standardized data-abstraction forms. The following characteristics were extracted: study characteristics included institution, publication year, first author, country, patient enrollment period, design (prospective or retrospective), number of patients, and reference standard, and clinical and radiologic characteristics included patient FIGO stages, histology of the tumor, age, lymph node metastasis, tumor size, treatment, 3-year overall survival (OS), disease-free interval (DFI), true positive, false positive, true negative, false negative, specificity, and sensitivity. 2 × 2 contingency tables were constructed, and we calculated the specificity, sensitivity, and likelihood ratios (LR). By using the Quality Assessment of Diagnostic Accuracy Studies 2 (QUADAS-2) tool, the reviewers assessed the methodological quality scores of the selected studies including 11 standard items, applying review manager software program (RevMan, version 5.0.2, Nordic Cochrane Centre, Copenhagen, Denmark). The answers “yes,” “no,” or “unclear” to the 11 standard questions represented that the risk of bias can be judged to be low, the potential for bias exists, or inadequate data are reported to permit a judgment, respectively.

### 2.3. Statistical Analysis

We analyzed the sensitivity, specificity, and accuracy of two diagnostic methods by Meta-DiSc 1.4 and Stata 15 with their 95% confidence intervals (CI). Also, we have drawn the hierarchical summary receiver operating characteristic (HSROC) curves. The random effect model or fixed effect model was used to evaluate the effect values based on the results of the heterogenicity test.

## 3. Results

**Figure 1:**
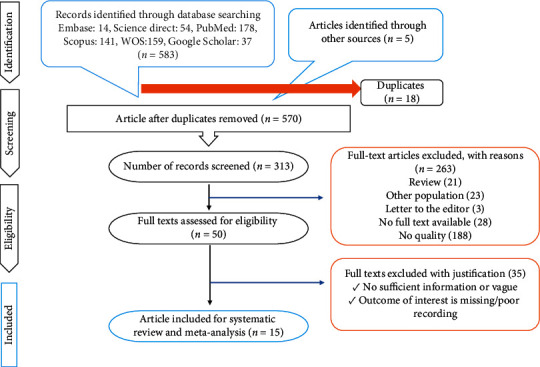
PRISMA flow diagram.

After a comprehensive computerized search, searching for articles and selecting them were done, and reference lists were cross-checked, as well. We recorded 1,435 files, of which 347 duplicate abstracts were removed. On the other hand, 641 unrelated studies, 89 conference abstracts, 73 case reports, 16 editorials, 27 letters, and 217 review articles were excluded. The remaining 25 full-text articles were examined for eligibility, and 7 articles were excluded (lack of required data to calculate the sensitivity and specificity of 18F-FDG PET/CT and MRI) to predict tumor response in LACC after CRT. Finally, 15 studies were selected to be included, and in the screening process of the references of these articles, no other eligible studies were found [20–37]. The features of the included studies are presented in Table 2. The detailed method of study selection in the current meta-analysis is shown in Figure 1.

### 3.1. Literature Review

#### 3.1.1. PET/CT

Rufini et al., Oh et al., and Choi et al., in their studies, focused on the correlation between the results of PET/CT and pathologic complete response. They suggested following the changes through the treatment process using delta SUV and delta TLG in order to achieve the most accurate diagnosis [14, 15, 20]. Choi et al. also suggested SUV_max_ of 4.0 as an optimal cutoff on the posttreatment PET/CT [15]. Scarsbrook et al. defined a five-point qualitative response assessment scoring system using which they reached a high sensitivity for PET/CT (28).

#### 3.1.2. MRI

Gui et al. reported a relatively low sensitivity and specificity for MRI. Their results indicated that MRI performance is not sufficient in distinguishing post-CRT inflammation from the residual tumor which can lead to a high number of false positives. However, the negative predictive value of MRI was high with a low risk of false negative [25]. The results of Atstupėnaitė were inline with this study and indicated high specificity and low sensitivity for MRI in post-CRP evaluation of patients [26].

#### 3.1.3. PET/CT and MRI

Perron et al. evaluated PET/CT and MRI accuracies in the same population. They reported a significantly higher Cohen Kappa coefficient between follow-up findings and PET/CT results compared to the findings of MRI. However, Vandecasteele et al., in their study on 27 cervical cancer patients, indicated a very low sensitivity for PET/CT and considered MRI as the preferred modality for recurrence assessment as it provided a specificity of 100% associated with a 74% NPV in their study [20, 21]. However, this difference could be caused by the small sample size in Vandecasteele et al.'s study. Su et al., on the other hand, confirmed the superiority of PET/CT in post-CRP patients (*P*=0.025) [24] (Table 1).

### 3.2. Study Description, Quality, and Publication Bias

All data analyses were performed on per-patient data. Out of 15 studies, 2 studies were done prospectively and 13 studies were retrospective analysis. A total of 1308 patients aged between 22 and 90 years were included in this study. The overall prevalence of patients with FIGO IB stage is 4.2% (95% CI: 2.4–6, *I*^2^: 90.3%) and FIGO IIA-IIB and IIIA-IIIB stages are 71.5% (95% CI: 67.4–75.6, *I*^2^: 85.1 2) and 14.7% (95% CI: 4/11–18), respectively. The staging data were only provided in 5 articles including 486 patients (17–19, 22, 23). The histopathology of 87.1% (95% CI: 87.1–84.4, *I*^2^:46.7%) of tumors was reported to be squamous cell carcinoma, and 12.6% (95% CI: 6.6–15.7, *I*^2^: 55.8%) tumors were adenocarcinomas. The histopathologic data were only provided in 6 articles including 526 patients (17–20, 22, 23). The main characteristics of 16 studies in a meta-analysis are given in Tables 2 and 3.

### 3.3. Methodological Quality Assessment

**Figure 2:**
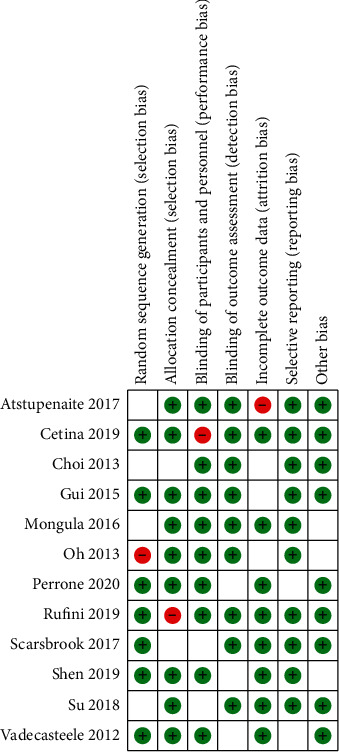
QUADS-2 for the evaluation of methodological quality.

Figure 2 indicates that the risk of bias applicability concerns summary of included studies, and in general, the quality of studies included is considered satisfactory.

### 3.4. Diagnostic Accuracy of PET/CT and MRI for Predicting the Tumor Response

**Figure 3:**
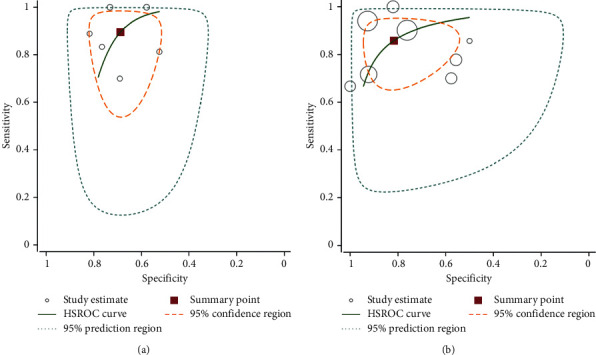
Hierarchical summary receiver (HSROC) curve for MRI (a) and 18F-FDG PET/CT (b) for predicting treatment response in LACC patients after CRT.

**Figure 4:**
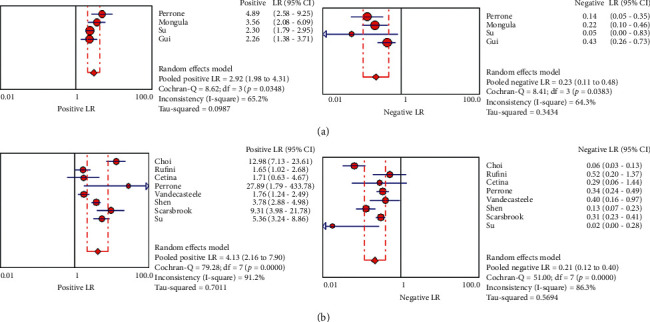
PLR and NLR of 18F-FDG PET/CT (b) and MRI (a) for predicting treatment response in LACC patients after CRT based on countries.

**Figure 5:**
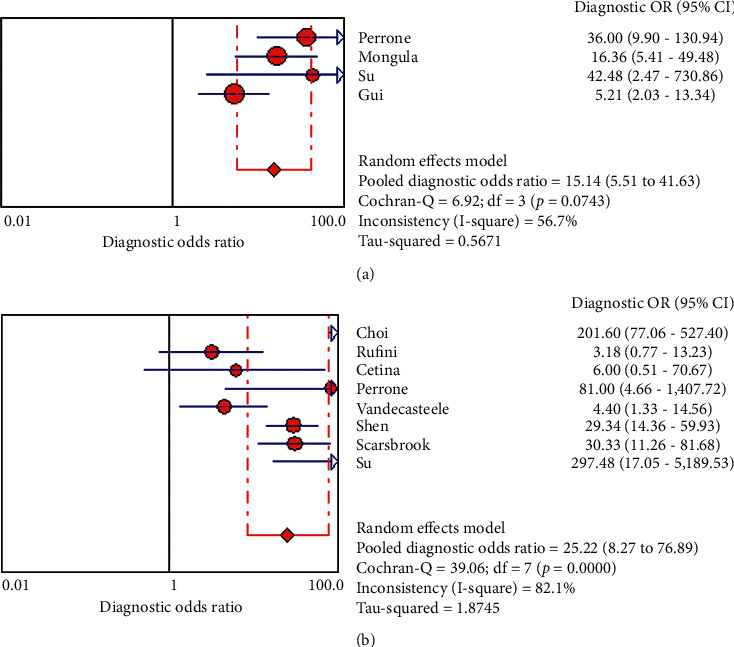
DOR of 18F-FDG PET/CT (b) and MRI (a) for predicting treatment response in LACC patients after CRT based on countries.

Overall diagnostic sensitivities of PET/CT and MRI for predicting the tumor response in locally advanced cervical carcinoma treated by chemoradiotherapy were 82.7% (95% CI: 75–88.6, *I*^2^: 71.9%) and 83.5% (95% CI: 79.9–86.6, *I*^2^: 86.7%), and the corresponding rates for specificities were 68.4% (95% CI: 62–74.4.1, *I*^2^: 70.7%) and 77.8% (95% CI: 74.4–81.1, *I*^2^: 91.2%), respectively. The DOR, PLR, and NLR for MRI were 15.140% (95% CI: 5.507–41.62, *I*^2^: 56.7%), 2.92 (95% CI: 1.980–4.305, *I*^2^: 65.5%), and 22.6 (95% CI: 10.6–48.2, *I*^2^: 64.3%). PET/CT had a DOR of 25.21 (95% CI: 8.27–76.88, *I*^2^: 82.1%). The PLR and NLR for PET/CT were 4.13 (95% CI: 2.16–7.89, *I*^2^: 91.2%) and 0.215 (95% CI: 0.116–0.398, *I*^2^: 86.3%), respectively (Tables 3 and 4, Figures 3–5). These ratios were obtained from 15 studies which consisted of 1132 patients.

### 3.5. Diagnostic Accuracy of PET/CT and MRI for the Detection of Residual Tumor

The diagnostic sensitivity and specificity of PET/CT for the detection of residual tumor in patients with locally advanced cervical carcinoma were 86% (95% CI: 83–0.90, *I*^2^: 92.8%) and 95.5% (95% CI: 93.4–98, *I*^2^: 96.3%), and the corresponding rates for MRI were 73.5% (95% CI: 69.3–78, *I*^2^: 87%) and 96.2% (95% CI: 94–98, *I*^2^: 94.6%), respectively. These data were according to 3 [26, 28, 29] and 4 [26, 29–31] articles including 293 and 290 patients for PET/CT and MRI.

### 3.6. Diagnostic Accuracy of PET/CT and MRI for the Detection of Tumor Metastases

The diagnostic sensitivity and specificity of PET/CT for the detection of tumor metastases in patients with locally advanced cervical carcinoma were 97% (95% CI: 94–99, *I*^2^: 97.3%) and 99% (95% CI: 96–100, *I*^2^: 00.0), and the corresponding rates for MRI were 31% (95% CI: 23–39 *I*^2^: 97.7%) and 98% (95% CI: 96–100 *I*^2^: 65.3%) based on 2 articles including 95 patients [20, 29].

### 3.7. Prevalence of Residual Disease, Pelvic Lymph Node Metastases, and Distant Metastases

The results of our meta-analysis revealed a prevalence of 12.7% (95% CI: 9.3–16, *I*^2^: 35.9%) for residual disease based on 4 articles including 378 patients [17, 20, 22, 23], a prevalence of 49% (95% CI: 43.7–54.3, *I*^2^: 81.3%) for pelvic lymph node metastases based on 4 articles including 330 patients [18, 20, 22, 23], and a prevalence of 11% (95% CI: 7.9–14.1, *I*^2^: 83.8%) for distant metastases in patients with locally advanced cervical cancer after chemo-radiotherapy based on 5 articles including 379 patients [17, 18, 20, 22, 23] (Table 3).

### 3.8. Meta-Analysis of 3-Year Overall Survival, PFS, and Mean DFI

The overall 3-year survival, PFS, and mean DFI in patients with locally advanced cervical cancer after chemoradiotherapy were 92.4% (95% CI: 87.3–97.6, *I*^2^: 0.8%) and 74.6% (95% CI: 66.1–83.1, *I*^2^: 0.0), respectively. The mean DFI for these patients was 15 months. Each of these ratios was obtained from 2 studies involving 100, 100, and 150 patients, respectively [18–23] (Table 3).

### 3.9. Meta-Analysis of SUV_mean_ in Patients with Complete and Partial Response

The overall SUV_mean_ for predicting treatment response in patients with locally advanced cervical cancer was reported to be 1.9 for patients who developed complete response and 5.10 for patients who did not, based on 4 articles including 372 patients [17–19, 22] (Table 3).

### 3.10. Fagan's Nomogram for the Calculation of Posttest Probabilities

**Figure 6:**
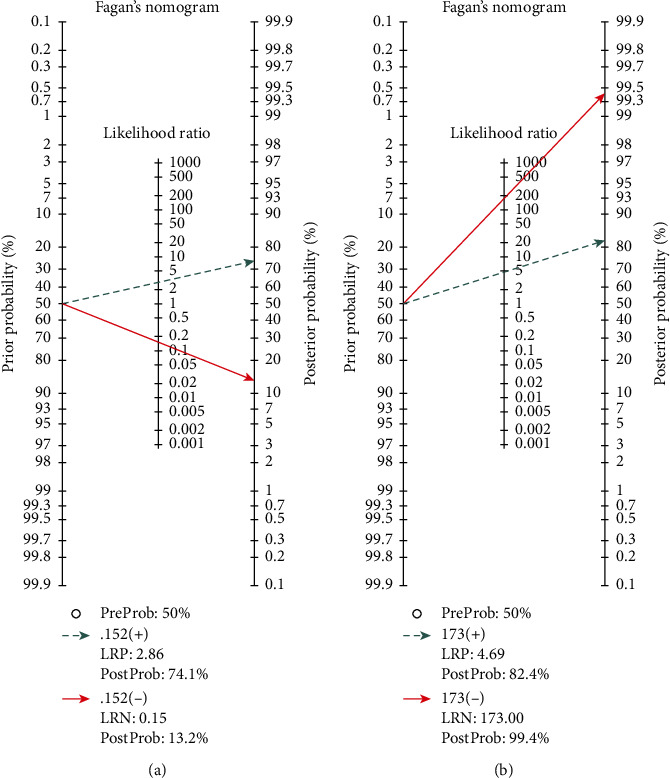
Fagan's nomogram for the calculation of posttest probabilities of MRI (a) and PET/CT (b).

A pretest probability of 50% for all three diagnostic tools was fixed, which was estimated by the number of patients in selected studies. In Figures 4 and 6, MRI had a posttest probability of 74.1% (a). For PET/CT (b), the posttest probability was 82.4%. If this patient tests positive, the posttest probability that she truly has developed residual disease or metastases would be 13.2% for MRI (a) and 99.4 for PET/CT (b) (solid line in red). The results were obtained by the following calculations: pretest odds = prevalence/1 − prevalence; posttest odds = pretest odds × LR − (LR+); posttest probability = posttest odds/1 + posttest odds (Figures 4 and 6).

## 4. Discussion

Among the most common cancers in women, cervical cancer is the third malignant tumor worldwide after breast and colorectal cancer. Cervical cancer has a significant impact on women's health because of its younger onset age, high prevalence, and posttreatment recurrence. The prevalence of residual disease and lymph node metastasis after chemotherapy were 12.7% and 49%, respectively. Predictors of recurrence of cervical cancer include the posttreatment tumor response as well as the status of the lymph nodes and clinical stage when it is diagnosed, initially [29–31]. Patients with no significant tumors after treatment are reported to have a 5-year survival of 76%. However, it has been reported that this rate is lower in patients who have findings which are suggestive of a tumor or who are diagnosed with persistent tumor (42% and 8%, respectively) [32]. According to the results of the present study, the overall 3-year survival for patients undergoing CRT was 92.4%. Due to the lack of data in the studies, we could not perform a subgroup analysis to divide patients into two groups with a complete response and partial response. However, we evaluated the overall DFI and PFS of these patients, which showed that the overall DFI was 74% and the mean PFS was evaluated to be 15 months. In cervical cancer, the surgeon's goal is to rule out the progression of the disease rather than macroscopically invasive because this treatment is not selective on parameters, but is provided by surgery. In cervical cancer, the role of pretreatment 18F-FDG-PET/CT and MRI is well defined. In case of suspected involvement in bladder, cervix, vagina, or rectum, MRI performs better [33, 34]. However, in the diagnosis of lymph node metastases or mesenteric, peritoneal, gastrointestinal, mediastinal, and pleural involvement, 18F-FDG-PET/CT is a more sensitive diagnostic tool [35]. Therefore, both of these diagnostic tools seem to be useful in choosing the treatment and in radiation therapy planning [36, 37]. However, studies assessing the response to chemotherapy are few, although this evaluation plays an important role in deciding on subsequent treatments [18, 39, 40]. Clinical and radiological features are required to diagnose residual disease. Unfortunately, it is difficult to perform gynecological examinations after radiation therapy. In fact, the accurate visualization of the cervix is interfered by vaginal adhesions and fibrosis after radiation [41]. MRI after radiotherapy may not assess the response, accurately due to heterogeneous gad-contrast enhancement and inflammation in areas which are hyperintense in T2W [42]. The evaluation of 18F-FDG-PET/CT can also be interfered by the inflammation and necrosis caused by radiotherapy [43]. Therefore, it is required to define the roles of these imaging modalities after chemoradiotherapy. In this meta-analysis, we assessed the role of 18F-FDG-PET/CT and MRI in predicting tumor response in LACC after CRT. This meta-analysis includes a total of 15 studies. Due to the fact that most individual studies have a limited number of cases, more data can be used in meta-analysis and also more reliable results are provided. Our study indicated better diagnostic sensitivity for MRI follow-up data (0.86) compared to PET/CT (0.83) in assessing the response to chemoradiotherapy for primary tumor and distant metastasis. However, the sensitivity of these two methods is quite similar, and there is no statistically significant difference between them. We also performed a subgroup analysis to decrease heterogeneity assessing the accuracy of these two methods among patients with residual disease compared to patients with distant metastases. MRI showed a real poor sensitivity in the detection of metastases (31% vs. 97%), and PET/CT performed significantly better. However, the difference among these two methods in the detection of residual disease was not significant (73% vs. 86%). Woo et al., in their meta-analysis, reported 73% sensitivity and 93% specificity for MRI in the diagnosis of parametric lesions in patients with cervical cancer [44], and Sakurai et al. declared that metabolic activity and standardized uptake value (SUV) depend on the tumor lesion (>1 cm); SUV was reported to have an average of 3.90 and 2.31 in tumoral and nontumoral lesions, respectively (*P* > 0.05) [45]. In our study, the mean SUV was 1.9 for patients who had a complete response and 10.10 for those who did not. Rufini et al. concluded that it is possible to evaluate the metabolic changes driven by treatment by performing 18F-FDG-PET/CT before, after, and during treatment in patients with LACC and assessing delta SUV parameters. However, they showed that the final assessment was not accurate enough to predict the pathological CR of the primary tumor [16]. Another study examined 25 patients with LACC (stages IB2–IIIB), evaluated them using both MRI and 18F-FDG PET/CT, before and after CRT, and indicated that 18F-FDG PET/CT provided important information which led to treatment planning changes in half of the patients. However, MRI detected the pelvic tumors in 2 patients which were missed by 18F-FDG-PET/CT [42]. In contrast, a meta-analysis of 15 studies represented by Maedes et al. assessed the additional diagnostic accuracy of 18F-FDG PET/CT in the whole body compared to conventional imaging in women with suspected recurrent/persistent cervical cancer. He reported that using 18F-FDG PET/CT in these patients was not supported by previous studies. However, the included studies in his meta-analysis had not compared PET/CT with MRI or CT [46]. This meta-analysis had several limitations. Firstly, not all studies in this meta-analysis reported the specific techniques applied. Some scanning parameters may affect the accuracy of PET/CT and MRI. Secondly, due to the small number of published articles in this field, there is a possibility of bias in the present study. Thirdly, as there were not enough articles comparing these modalities in the same sample size, we had to include articles evaluating one of the modalities and then compare their results in a meta-analysis. This can be our major source of bias. Finally, the number of patients in the included studies was relatively small, which may lead to bias in the final results.

## 5. Conclusion

18F-FDG PET/CT seems to have a better overall diagnostic accuracy in the evaluation of the treatment response to chemotherapy in LACC patients. MRI showed a really poor sensitivity in the detection of metastases, and PET/CT performed significantly better. However, the difference between these two methods in the detection of residual disease was not significant. More studies need to be conducted in order to approve that 18F-FDG PET/CT can be a standard option to assess the treatment response.

## Figures and Tables

**Table 1:** The required data for construction of a 2 × 2 table.

Author	TP	TN	FP	FN	Imaging method
Choi et al. [15]	63	64	5	4	PET/CT
Rufini et al. [16]	30	29	11	18	PET/CT
Cetina et al. [17]	12	2	2	0	PET/CT
Perrone et al. [18]	20	10	10	0	PET/CT
	16	18	4	2	MRI
Vandecasteele et al. [19]	2	11	9	1	PET/CT
Oh et al. [20]	8	40	0	12	PET/CT
Shen et al. [21]	15	65	21	6	PET/CT
Mongula et al. [22]	15	18	6	3	MRI
Scarsbrook et al. [23]	45	30	6	3	PET/CT
Su et al. [24]	16	27	0	6	PET/CT
	7	27	0	20	MRI
Gui et al. [25]	2	27	11	1	MRI
Atstupėnaite et al. [26]	16	26	0	9	MRI

**Table 2:** Study characteristics.

Author	Year	Duration	Country	*N*	Mean age	Study design
Choi et al. [15]	2013	2005–2012	Korea	136	57.1	Retro
Rufini et al. [16]	2019	2010–2014	Italy	88	49.5 (22–75)	Pros
Cetina et al. [17]	2011	2007–2008	Mexico	60	47.2	Retro
Perrone et al. [18]	2020	2007–2017	Italy	40	55	Retro
Vandecasteele et al. [19]	2012	2007–2010	Belgium	27	49	Retro
Chen et al. [38]	2018	2009–2015	Taiwan	142	55	Retro
Oh et al. [20]	2013	2009–2010	Korea	60	53.5	Retro
Lucia et al. [27]	2017	2010–2016	France	102	58	Retro
Miccò et al. [28]	2014	2009–2012	USA	49	45	Retro
Shen et al. [21]	2019	2019–2015	Taiwan	142	55	Retro
Mongula et al. [22]	2016	—	—	42	53	Rero
Scarsbrook et al. [23]	2017	2011–2014	UK	96	47	Retro
Su et al. [24]	2018	—	Taiwan	55	—	Retro
Gui et al. [25]	2015	2005–2008	Italy	41	48	Pro
Atstupėnaite et al. [26]	2017	2009–2010	Lithuania	52	—	Retro

**Table 3:** Patients' pathologic and imaging characteristics.

Author	FIGO stage			Histopathology		Pelvic LN metastasis	Prevalence of distant metastasis	3-OS	DFI (month)	MTV	TLG	Persistence	CR	PFS	SUVmean	
	IB	IIA-IIB	IIIA-IIIB	SCC	Adenocarcinoma										Partial response	Complete response
Rufini et al.	3 (3.4%)	72 (81.7%)	13 (14.7%)	77 (87.5%)	11 (12.5%)	40 (45.5%)	10 (11.4%)	N/A	15.5	33.8 (1.2–180.0)	240.2 (7.9–2417.0)	N/A	N/A	N/A	2.1 (1.1–5.7)	1.8 ± 0.7
Cetina et al.	2/16 (12%)	8/16 (50%)	3/16 (19%)	15/16-(94%)	1 (6.2%)	N/A	N/A	N/A	14.5	N/A	N/A	N/A	N/A	N/A	N/A	N/A
Perrone et al.	N/A	N/A	N/A	32 (80%)	8 (20%)	17 (42.5%)	1 (2.5%)	88%	N/A	N/A	N/A	8 (20%)	33 (82.5%)	78%	7.5 ± 5.1	N/A
Chen et al.	37 (26%)	78 (55%)	27 (19%)	114 (80%)	28 (20%)	61 (43%)	27 (19%)	N/A	N/A	28.1 ± 42.7	216.1 ± 280.5	22 (15%)	120 (84%)	N/A	5.12 ± 1.17	1.28
Choi et al.	3 (1.9%)	102 (75%)	17 (12.4%)	124 (91.2%)	9 (6.6%)	N/A	12 (22.2%)	N/A	N/A	N/A	N/A	18 (13.2%)	124 (91%)	N/A	5.7 ± 2.6	2.5 ± 0.8
Oh et al.	3 (5%)	46 (77%)	8 (13%)	52 (87%)	8 (13%)	42 (70%)	6 (10%)	93.6%	N/A	N/A	N/A	4 (7%)	43 (73%)	71.9%	N/A	N/A

SCC: squamous cell carcinoma; LN: lymph node; DFI: disease-free interval; 3-OS: 3-month overall survival; MTV: metabolic tumor volume; TLG: total lesion glycolysis; CR: complete response; PFS: progression-free survival; and SUV: standardized uptake value.

**Table 4:** Diagnostic performance of MRI and PET/CT in patients with LACC post-CRT.

Parameter	PET/CT	MRI
Sensitivity (95% CI)	0.835 (CI 95% 0.799–0.866)	0.827 (CI 95% 0.75–0.88)
Specificity (95% CI)	0.778 (CI 95% 0.744–0.811)	0.684 (CI 95% 0.620–0.744)
PLR (95% CI)	4.135 (CI 95% 2.164–7.899)	2.920 (CI 95% 1.980–4.305)
NLR (95% CI)	0.215 (CI 95% 0.116–0.398)	0.226 (CI 95% 0.106–0.482)
DOR (95% CI)	25.216 (CI 95% 8.270–76.887)	15.140 (CI 95% 5.507–41.626)
PPV	75%	85%
NPV	62%	86%
Accuracy	80%	74%
AUC-SROC	0.8007	0.9081
*I* ^2^	65%	82%
*Q* index	0.7384	0.8379

## Data Availability

The data used to support the findings of this study are available from the corresponding author upon request.

## Abbreviations

18F-FDG-PET/CT: 18F-fluorodeoxyglucose positron emission tomography/computerized tomography
MRI: Magnetic resonance imaging
LACC: Locally advanced cervical cancer
CRT: Chemoradio therapy
QUADAS-2: Quality Assessment of Diagnostic Accuracy Studies 2
DOR: Diagnostic odds ratio
PLR: Positive likelihood ratio
NLR: Negative likelihood ratio
CT: Computed tomography
PET: Positron emission tomography
DFI: Disease-free interval
PFS: Progression-free survival
OS: Overall survival
CI: Confidence interval
SUV: Standardized uptake value.
